# The microRNA-200 family targets multiple non-small cell lung cancer prognostic markers in H1299 cells and BEAS-2B cells

**DOI:** 10.3892/ijo.2013.1963

**Published:** 2013-05-27

**Authors:** MARICICA PACURARI, JOSEPH B. ADDISON, NAVEEN BONDALAPATI, YING-WOOI WAN, DAJIE LUO, YONG QIAN, VINCENT CASTRANOVA, ALEXEY V. IVANOV, NANCY LAN GUO

**Affiliations:** 1 Mary Babb Randolph Cancer Center, West Virginia University, Morgantown, WV 26505, USA; 2 Department of Biochemistry, School of Medicine, West Virginia University, Morgantown, WV 26505, USA; 3 School of Public Health, West Virginia University, Morgantown, WV 26505, USA; 4 Pathology and Physiology Research Branch, Health Effects Laboratory Division, National Institute for Occupational Safety and Health, Morgantown, WV 26505, USA

**Keywords:** non-small cell lung cancer, miR-200 family, epithelialmesenchymal transition, prognostic biomarkers, metastasis

## Abstract

Lung cancer remains the leading cause of cancer-related mortality for both men and women. Tumor recurrence and metastasis is the major cause of lung cancer treatment failure and death. The microRNA-200 (miR-200) family is a powerful regulator of the epithelial-mesenchymal transition (EMT) process, which is essential in tumor metastasis. Nevertheless, miR-200 family target genes that promote metastasis in non-small cell lung cancer (NSCLC) remain largely unknown. Here, we sought to investigate whether the microRNA-200 family regulates our previously identified NSCLC prognostic marker genes associated with metastasis, as potential molecular targets. Novel miRNA targets were predicted using bioinformatics tools based on correlation analyses of miRNA and mRNA expression in 57 squamous cell lung cancer tumor samples. The predicted target genes were validated with quantitative RT-PCR assays and western blot analysis following re-expression of miR-200a, -200b and -200c in the metastatic NSCLC H1299 cell line. The results show that restoring miR-200a or miR-200c in H1299 cells induces downregulation of *DLC1*, *ATRX* and *HFE*. Reinforced miR-200b expression results in downregulation of *DLC1*, *HNRNPA3* and *HFE*. Additionally, miR-200 family downregulates *HNRNPR3*, *HFE* and *ATRX* in BEAS-2B immortalized lung epithelial cells in quantitative RT-PCR and western blot assays. The miR-200 family and these potential targets are functionally involved in canonical pathways of immune response, molecular mechanisms of cancer, metastasis signaling, cell-cell communication, proliferation and DNA repair in Ingenuity pathway analysis (IPA). These results indicate that re-expression of miR-200 downregulates our previously identified NSCLC prognostic biomarkers in metastatic NSCLC cells. These results provide new insights into miR-200 regulation in lung cancer metastasis and consequent clinical outcome, and may provide a potential basis for innovative therapeutic approaches for the treatment of this deadly disease.

## Introduction

Lung cancer remains the leading cause of cancer-related mortality in the world, with an overall 5-year survival rate of 15%. Approximately 85% of lung cancer cases are non-small cell lung cancer (NSCLC) (1). Tumor recurrence and metastasis is the major cause of lung cancer treatment failure and death. Epithelial-mesenchymal transition (EMT) plays an important role in tumor progression and is a key feature of metastasis in many cancer types, including lung cancer (2,3). EMT characteristics include perturbations of several signaling pathways including the loss of E-cadherin expression, which is a major step in metastasis of NSCLC (4).

In the past few years, microRNAs (miRNAs) have emerged as promising molecular factors with potential for clinical applications in cancer diagnosis and therapy (5–8). MicroRNAs are small endogenous non-coding RNAs that range 19–24 nucleotides in length. MicroRNAs regulate the expression of numerous genes either via translational silencing or by inducing mRNA degradation of the targeted genes (5). Moreover, it has been estimated that one miRNA can modulate as many as 200 genes, and over 30% of human coding genes are under miRNA regulation (9,10). Increasing evidence indicates that in human cancers, miRNAs can act either as oncogenes or as tumor suppressor genes (11,12). To date, more than 1,400 miRNA sequences have been identified in human cells (13).

The microRNA-200 (miR-200) family, represented by miR-200a, -200b, -200c, -141 and -429, is a marker and powerful regulator of the EMT process. Its functions include maintaining the epithelial phenotype of tissues through suppression of the EMT-inducing transcription factors zinc finger E-box binding homeobox 1 and 2 (ZEB1 and ZEB2) (14,15). It has been shown that suppression of ZEB1 in undifferentiated mesenchymal-like cells leads to restoration of epithelial phenotype with increased expression of epithelial phenotype marker, E-cadherin (16), which mediates cell-cell adhesion (14). In many cancer types, E-cadherin is somatically inactivated via mutation, truncation or epigenetic silencing, a loss that enables the cells to acquire a highly invasive phenotype with the characteristics of EMT (17). Recent studies have shown that restoring miR-200c expression decreases cell migration but does not result in E-cadherin re-expression in some cells, thus suggesting that miR-200c targets other genes involved in tumor progression and metastasis (18).

The goal of this study is to identify potential targets of miR-200 family essential in NSCLC metastasis and clinical outcome. Our previous studies identified prognostic biomarkers associated with metastasis in early stage NSCLC tumors not treated with chemotherapy (19–21). Specifically, a 35-gene signature was identified (19) and validated (20) as predictive of tumor metastasis in 434 NSCLC patients, including lung adenocarcinoma, squamous cell lung cancer and large cell lung cancer. This signature could identify more aggressive tumors from stage IA NSCLC (20). In another genome-wide DNA microarray analysis of data from the Director’s Challenge study (22), 12- and 15-gene prognostic signatures were identified and validated using multi-center NSCLC patient cohorts (n=442) (21). All the identified prognostic biomarkers were confirmed with quantitative RT-PCR analysis and some were validated with western blot assays of independent snap-frozen human NSCLC tumors (20). Based on these results, we sought to identify key miRNAs that regulate multiple NSCLC prognostic marker genes, to reveal molecular regulatory events in metastasis with implications on clinical outcome. The following experimental analyses were carried out in this study. First, bioinformatics methods were used to predict miRNA regulators of the identified signature genes in NSCLC. Then, miRNAs that could potentially regulate multiple prognostic biomarkers were identified. Based on the bioinformatic prediction results, the miR-200 family was selected to further determine a regulation of the predicted target genes and to determine putative molecular networks in EMT and tumor metastasis.

## Materials and methods

### Patient samples and gene expression profiling

A total of 130 lung squamous cell carcinoma (SCC) samples were analyzed in this study. The patient characteristics were described in a previous publication (23). Genome-wide mRNA expression profiles of the tumor samples were quantified with the Affymetrix U133A Gene Chip (23). Microarray data were extracted and calculated using the Affymetrix MAS 5 software after global scaling of the average intensity to 600. The mRNA microarray data were available from Gene Expression Omnibus (GEO, http://www.ncbi.nlm.nih.gov/geo/) with accession no. GSE4573. Out of 130 SCC tumors, 57 samples were screened for miRNA expression profiles with TaqMan miRNA assays (Applied Biosystems Inc., Foster City, CA) (24). The miRNA expression data was available from GEO with accession no. GSE16025. The mRNA and miRNA profiles of these matched 57 tumor samples were used for further analysis.

### MicroRNA target prediction

The mRNA expression levels of the lung cancer prognostic markers identified in our previous studies (19–21) were retrieved from the genome-wide expression profiles of the 57 SCC tumor samples. Pearson’s correlation coefficient between each mRNA and all available miRNAs was computed. Significant mRNA-miRNA pairs (|r|≥0.258; p≤0.05) were selected for target prediction. Four Bioinformatics tools were used in the study for miRNA target prediction, including TargetScan (http://www.targetscan.org/) (10), PicTar (http://www.pictar.mdc-berlin.de/) (25), miRDB (http://www.mirdb.org/miRDB/) and microRNA.org (http://www.microrna.org/microrna/home.do) (26). These computational methods use sequence complementary base-pairing between miRNA and mRNA in target prediction. Files and databases containing miRNA and predicted target genes were downloaded from the online websites of these toolsets. These files were then analyzed with in-house software script written in R to identify miRNA-mRNA gene pairs showing significant expression correlation in SCC tumor samples. In addition, TarBase (http://www.diana.cslab.ece.ntua.gr/tarbase/) (27) was used to retrieve experimentally validated miRNA targets.

The miRNA targets predicted by TargetScan are based on the presence of conserved 8mer, 7mer and 6mer sites that match the seed region of each miRNA (10). TargetScan also predicts non-conserved sites, additional types of seed matches that are preferentially conserved in different species, and sites with mismatches in the seed region that are compensated by conserved 3′ pairing (28). PicTar predicts miRNA targets by searching 3′ UTR alignments with predicted sites (25). The miRDB uses an algorithm, MirTarget2, based on Support Vector Machine, to predict miRNA targets. The microRNA.org uses miRanda algorithm to predict miRNA targets based on sequence and contextual features of the predicted miRNA-mRNA duplex (26).

### MicroRNA and mRNA binding sites

The binding sites between each miRNA-mRNA pair was retrieved from the *Homo sapiens* microRNA.org database with the ‘Target mRNA’ module. The searching parameters were set with mirSVR score ≤0 and PhastCons score ≥0.

### Cell lines and cell culture

Small airway epithelial cells (SAEC) and normal human bronchial/tracheal epithelial cells (NHBE) were obtained from Lonza Walkersville Inc. (Walkersville, MD). Human non-small cell lung cancer cell line H1299 and human immortalized lung epithelial cell line BEAS-2B were purchased from the American Type Culture Collection (ATCC, Manassas, VA). SAEC cells were cultured according to the supplier’s recommendations in SABM medium supplemented with 52 *μ*g/ml bovine pituitary extract, 0.5 ng/ml human recombinant epidermal growth factor (EGF), 0.5 *μ*g/ml epinephrine, 1 *μ*g/ml hydrocortisone, 10 *μ*g/ml transferrin, 5 *μ*g/ml insulin, 6.5 ng/ml triiodothyronine, 50 *μ*g/ml gentamicin/amphotericin B (GA-1000) and 50 *μ*g/ml fatty acid-free bovine serum albumin. NHBE cells were cultured according to the supplier’s recommendations in BEBM media supplemented with bovine pituitary extract (52 *μ*g/ml), hydrocortisone (0.5 *μ*g/ml), human epidermal growth factor (hEGF, 0.5 ng/ml), epinephrine (0.5 *μ*g/ml), insulin (5 *μ*g/ml), triiodothyronine (6.5 ng/ml), transferrin (10 *μ*g/ml), gentamicin (50 *μ*g/ml), amphotericin B (50 ng/ml), bovine serum albumin (1.5 *μ*g/ml). H1299 and BEAS-2B cells were cultured in Dulbecco’s modified Eagle’s medium (DMEM) supplemented with penicillin, streptomycin, L-glutamine and 10% fetal bovine serum. All cells were cultured at 37°C in humidified incubator with 95% air and 5% CO_2_.

### Virus transduction

Human miRIDIAN shMIMIC lentiviral miRNA particles (hsa-miR-200a: UAACACUGUCUGGUA ACGAUGU, hsa-miR-200b: UAAUACUGCCUGGUAAUG AUGA, hsa-miR-200c: UAAUACUGCCGGGUAAUGA UGGA, and control scrambled microRNA were purchased from Open Biosystems (Huntsville, AL) and used for infection of target cells in the presence of 4 *μ*g/ml of polybrene.

### Western blot analysis

Cells were lysed in 1X SDS lysis buffer (50 mM Tris-HCl, pH 6.8, 2% SDS, 10% glycerol). Total protein was quantified by the BCA method. β-mercaptoethanol was added to lysates to a final concentration 100 mM. Equal amounts of total protein were separated by 4–12% SDS-PAGE and transferred to a PVDF membrane. Membranes were blocked 1 h with 5% non-fat milk in 1X PBS containing 0.05% Tween-20. Membranes were then incubated for 1 h at room temperature with primary antibodies. After incubation with the primary antibody, membranes were washed thrice in 1X PBS with 0.05% Tween-20 for 5 min each and blocked for 7 min in blocking solution. Membranes were incubated for 1 h at room temperature with horseradish peroxidase (HRP) conjugated donkey anti-mouse IgG or donkey anti-rabbit IgG in 1X PBS with 0.05% Tween-20. Membranes were then washed five times for 5 min in PBS-Tween-20 and finally developed with HyGLO Western Blotting Substrate (Denville Scientific) according to the instructions of the manufacturer. Protein band intensity was determined using FluorChem^®^ Q software (AlphaInnotech, Santa Clara, CA). Relative protein level was determined after normalization to tubulin and relative to negative control (miR-scr) samples. The following antibodies were used: *ATRX* (Santa Cruz Biotechnology, catalog no. SC-55584), *DLC1* (BD Biosciences, catalog no. 612020), *HFE* (Santa Cruz Biotechnology, catalog no. SC-130375), *ZEB1* (Sigma, catalog no. HPA027524), *HNRNPA3* (Santa Cruz Biotechnology, catalog no. SC-133665), *E-cadherin* (BD Biosciences, catalog no. 610181), *GAPDH* (Millipore, catalog no. MAB374) and *tubulin* (Sigma, catalog no. T9026).

### RNA isolation

Total RNA was extracted using the mirVana^®^ kit (Ambion Inc., Austin, TX) according to the manufacturer’s protocol. To ensure a good RNA quality, the quality and integrity of the total RNA was evaluated using 28S/18S ratio and a visual image of the 28S and 18S bands were evaluated on the 2100 Bioanalyzer (Agilent Technologies, Santa Clara, CA). RNA isolated using this method yielded a very good quality, with a RIN number ≥9. Concentration of the total RNA was assessed using the NanoDrop-1000 Spectrophotometer (NanoDrop Technologies, Germany).

### Quantitative real-time RT-PCR

Complementary DNA (cDNA) was generated using total RNA according to the TaqMan^®^ MicroRNA Reverse Transcription protocol (Applied Biosystems Inc.). Quantitative RT-PCR for microRNA was performed using TaqMan MicroRNA assays (Applied Biosystems Inc.). Human U47 small nuclear RNA was used as an endogenous control. The expression levels of miRNAs were quantified using ABI 7500 quantitative real-time instrument and SDS software (Applied Biosystems Inc.). The abundance of miRNA is expressed as Ct (threshold fluorescence) which gives the number of cycles required to reach threshold fluorescence. Real-time PCR for target genes was determined using total RNA and cDNA was generated using a High-Capacity cDNA Reverse Transcription kit and TaqMan gene expression assays (Applied Biosystems Inc.). E-cadherin (CDH1) mRNA was measured using SYBR-Green Master mix and CDH1 specific primers according to manufacturer’s protocol (Applied Biosystems Inc.). All qRT-PCR reactions were performed on 7500 instrument (Applied Biosystems Inc.). In the qRT-PCR analysis of E-cadherin, the dissociation curve showed the absence of a secondary peak, indicating no presence of primer dimer. Specificity of the PCR product obtained from SYBR-Green reactions was verified by sequencing. The expression level of each gene was determined by following formulas: fold change = 2^−ΔΔCt^, where ΔCt (cycle threshold) = Ct_target gene_ - Ct_endogenous control gene_, and ΔΔCt = ΔCt_treated sample_ - ΔCt_control sample_. The expression level of the analyzed genes is reported as fold change relative to negative miR-scrambled (-src) infected samples. The human UBC gene was used as an endogenous control gene.

In this study, a predicted gene was considered a confirmed target if the mRNA level was significantly downregulated or the protein level was downregulated at least 15% relative to negative control samples. Not all of the predicted targets were analyzed at the protein level due to the lack of specificity of commercially available antibodies.

### Functional pathway analysis

Ingenuity pathway analysis (IPA) software (Ingenuity Systems, Redwood City, CA) was used to derive curated molecular interactions reported in the scientific literature. These interactions included both physical and functional interactions, as well as interactions representing pathway relevance. In this study, in order to delineate molecular networks of genes interacting with the miR-200 family and novel molecular targets, a core analysis was employed to identify the most relevant canonical pathways, biological functions and physiological processes from the interactions reported in the IPA database. We then selected pathways that were statistically significant with a p<0.05 in adjusted Benjamini-Hochberg tests.

### Statistical analysis

The statistical significance of the difference between groups was determined by un-paired t-tests at p≤0.05. The qRT-PCR expression data are presented as mean ± SEM.

## Results

### Prediction of miR-200 novel molecular targets

**Figure 1:**
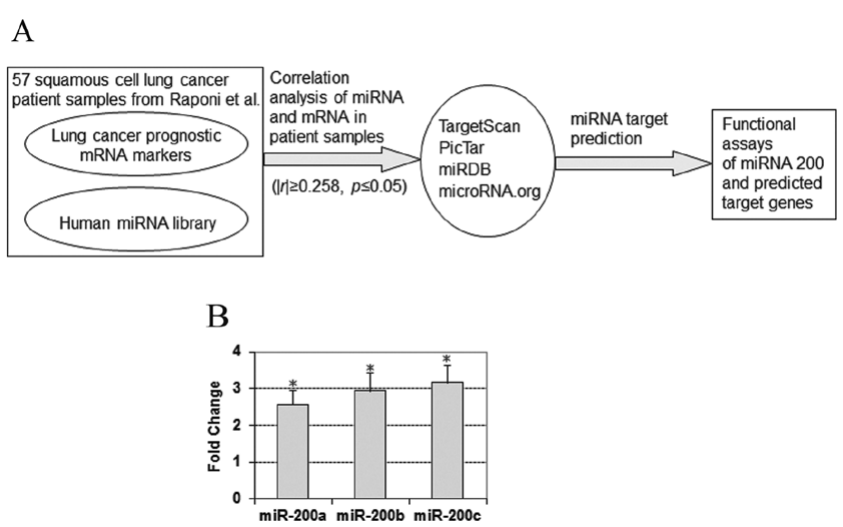
Computational prediction of miRNA and target lung cancer prognostic genes and experimental results focusing on miR-200 family. (A) Overview of bioinformatic prediction of miRNA target genes and functional assays. (B) Gene expression fold change of miR-200 in primary squamous cell lung cancer tumors vs. normal lung tissues in the patient cohort from Raponi *et al*(24). ^*^Statistically significant at p≤0.05. Data presented as mean ± SEM.

To screen for potential miRNA regulators of our previously identified lung cancer prognostic gene signatures (19–21), the correlation between the mRNA expression of these biomarkers and all available human miRNA expression profiles in 57 squamous cell lung cancer tumors was computed. For all miRNA-mRNA gene pairs showing significant correlation (|r|>0.258, p≤0.05, Pearson’s correlation analysis) in the lung SCC tumors, 4 bioinformatics toolsets (TargetScan, PicTar, miRDB and microRNA.org) were used to determine whether or not a given gene is a predicted target of the corresponding miRNA (Fig. 1A). A total of 233 miRNA-mRNA gene pairs were predicted as a target pair by at least one bioinformatic method. The correlation analysis and the prediction results may be viewed on our website (http://www.wvucancer.org/guoLab/Publications). Due to alternative splicing, each gene may have multiple probes in DNA microarray data. This will lead to discrepant gene expression among different probes for the same gene and discordant correlation between mRNA and miRNA. The mRNA-miRNA pairs with a negative correlation for at least one probe set were selected for further analysis.

Several miRNAs, including the miR-200 family, were predicted to target multiple prognostic biomarkers. We focused on the miR-200 family because of its reported role in tumor metastasis. The miRNA-200 family is represented by miR-200a, miR-200b, miR-200c, miR-141 and miR-429, based on their genomic location and primary sequence. Based on sequence similarity, the miR-200 family is divided into two subclasses: one class includes miR-200b, -200c and -429, and the other class includes miR-200a and miR-141 (29). The members in each subclass share the same seed sequence. We analyzed the expression levels of the miR-200s in squamous cell lung cancer patient primary tumors and normal lung tissues in the cohort from Raponi *et al*(24). The results show that miR-200a had a 2.56-fold overexpression (p<1.35e-7; unpaired t-tests) in the tumors, miR-200b exhibited a 2.94-fold overexpression (p<1.32e-6; unpaired t-tests) and miR-200c showed a 3.16-fold overexpression (p<0.001; unpaired t-tests) in the patient tumors (Fig. 1B). However, during metastasis, previous studies showed that the miR-200 family expression is lost in mesenchymal subtypes of epithelial cancers and negatively correlates with cancer cell invasion (15,16).

The predicted targets for hsa-miR-200a include deleted in liver cancer 1 gene (*DLC1*), E2F transcription factor 4 (*E2F4*), and *AHNAK* nucleoprotein (desmoyokin) (*AHNAK*); for hsa-miR-200b: *DLC1*, ubiquitin-like modifier activating enzyme 6 (*UBA6*), ubiquitin-conjugating enzyme E2I (*UBE2I*) and heterogeneous nuclear ribonucleoprotein A3 (*HNRNPA3*); for hsa-miR-200c: alpha thalassemia/mental retardation syndrome X-linked gene (*ATRX*), hereditary hemochromatosis (*HFE*), *DLC1* and thrombospondin 1 (*THBS*) (Tables I and II). To confirm the regulation of miR-200a, -200b, -200c on the predicted targets, expression of these prognostic biomarkers genes at the mRNA and protein levels were examined following re-expression of miR-200a, -200b, -200c in H1299 and BEAS-2B cells.

### Restoring miR-200 expression in H1299 cells

**Figure 2:**
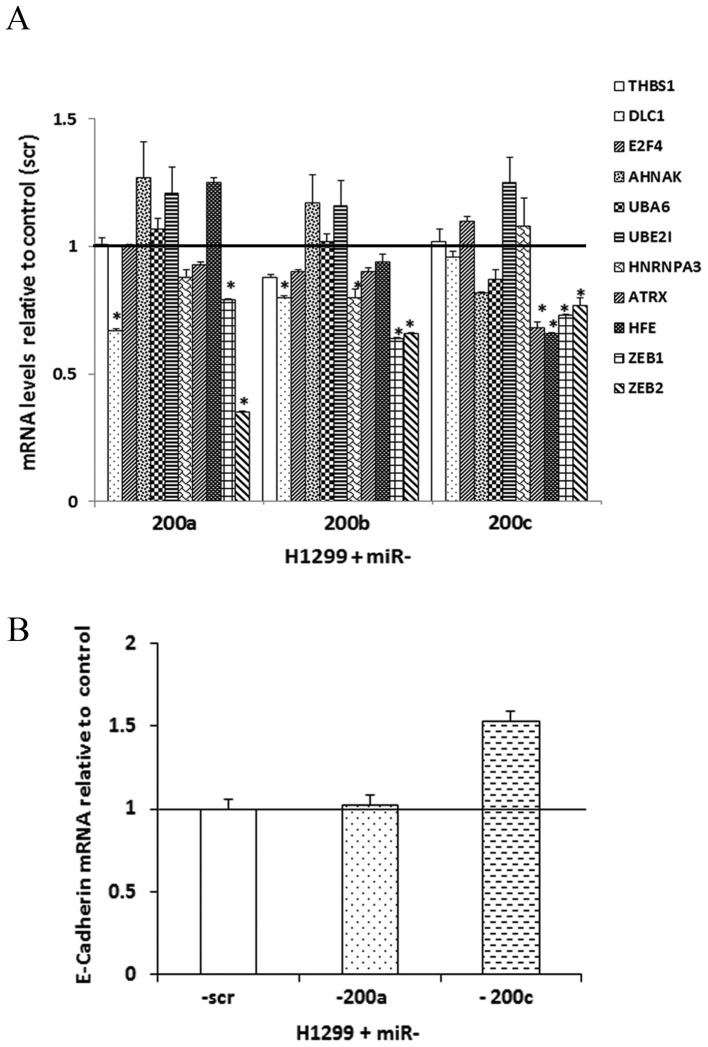
Relative mRNA levels of the predicted targets of miR-200 in H1299. (A) Relative mRNA expression of predicted miR-200 target genes in H1299 cells infected with miR-scrambled and miR-200a, -200b and -200c. These targets were predicted with microRNA.org based on 3′-UTR and downregulation after microRNA transduction. (B) Relative mRNA expression level of E-cadherin in H1299 infected with miR-200a and -200c. Cells infected with miR-scr were used as a negative control. The mRNA expression levels were determined using qRT-PCR as described in Materials and methods. ^*^Statistically significant at p≤0.05, n=3 (biological replicates). Data presented as mean ± SEM. Relative mRNA level was calculated after normalization to endogenous gene, UBC and relative to negative control, miR-scr.

We analyzed the expression levels of miR-200a, -200b -and 200c in a metastatic human NSCLC model, H1299 cells. Normal human small airway epithelial cells (SAEC) were used as control cells. The expression level of miR-200a, -200b and -200c in H1299 cells was at the detection limit (Fig. 2). In contrast, SAEC expressed higher levels of miR-200a, -200b and -200c (data not shown; http://www.wvucancer.org/guoLab/Publications) consistent with their normal epithelial phenotype. In order to identify potential molecular targets of miR-200a, -200b, -200c, H1299 cells were stably infected with lentiviral vectors expressing miR-200a, -200b, -200c or negative control miRNAs. The infected H1299 cells expressed high levels of exogenous miR-200a, -200b, -200c, which were comparable with the levels exhibited in normal lung epithelial SAEC cells (data not shown; http://www.wvucancer.org/guoLab/Publications).

### miR-200 regulation on predicted molecular targets

*ZEB1* and *ZEB2* genes are the most extensively characterized targets of the miR-200 family (14,15) and they were used as positive controls in the qRT-PCR experiments. In H1299 cells over-expressing miR-200a, -200b and -200c, *ZEB1* and *ZEB2* exhibited significant (p<0.05; unpaired t-tests) downregulation at the mRNA expression level (Fig. 2A). Following overexpression of miR-200a, *DLC1* showed significantly decreased mRNA expression. Restoring miR-200b resulted in significant downregulation of *DLC1* and *HNRNPA3* at the mRNA level. The overexpression of miR-200c resulted in a significant mRNA downregulation of *ATRX* and *HFE* (Fig. 2A).

**Figure 3:**
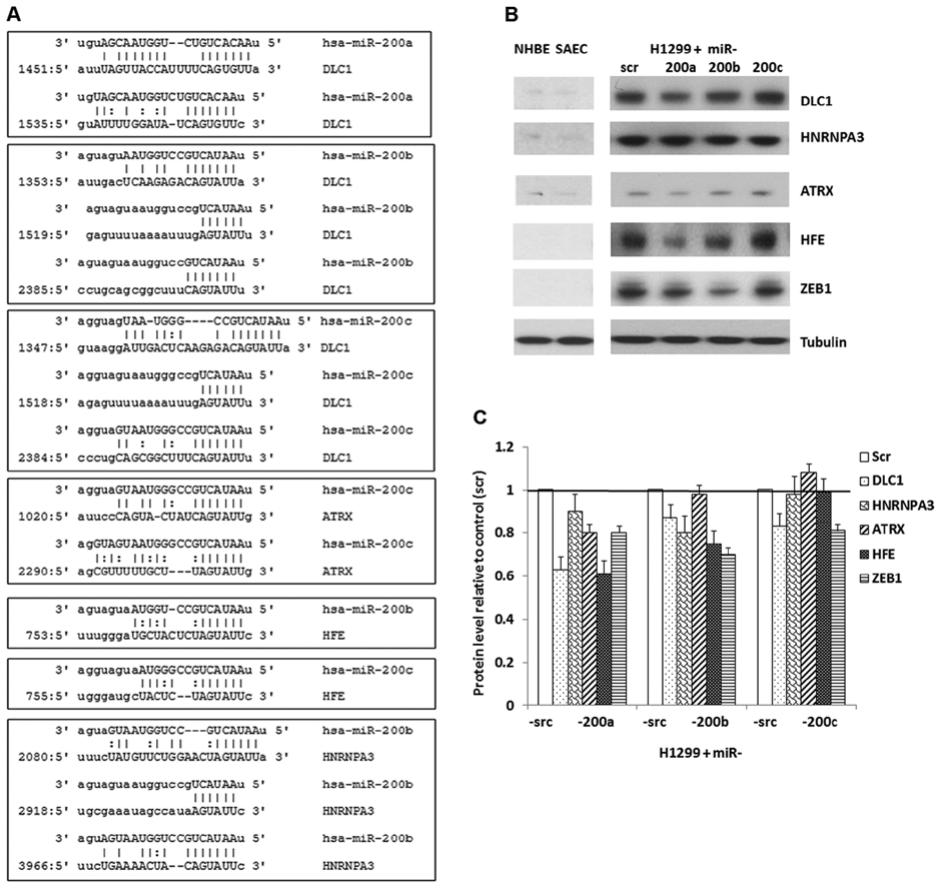
Protein levels of the predicted molecular targets of miR-200 in H1299 cells after infection with miR-scr (scrambled) or with miR-200a, -200b or -200c. miR-scr was used as a negative control. (A) 3′-UTR sequences of the miR-200a, -200b and -200c putative binding sites of target genes is given in the 5′- to 3′-orientation. (B) Western blot analysis of protein levels in H1299 cells over-expressing miR-200a, -200b, -200c or miR-scr. One representative blot is shown. The experiments were repeated in three biological replicates. Tubulin was used as a loading control. (C) Semi-quantitative analysis of protein levels relative to negative control miR-scr. Protein level was determined as described in Materials and methods.

At the protein level, *ZEB1* was reduced in H1299 cells overexpressing miR-200a, -200b and -200c, with the most downregulation (about 30%) in cells re-expressing miR-200b (Fig. 3B and C). A considerable downregulation of *DLC1*, *ATRX* and *HFE* was observed in H1299 cells overexpressing miR-200a. It is noteworthy that *DLC1* and *HFE* had about 40% downregulation in H1299 cells infected with miR-200a mimic, exhibiting a more significant downregulation than *ZEB1*. In H1299 cells with restored expression of miR-200b, the protein levels for *DLC1*, *HNRNPA3* and *HFE* were reduced by 15–30%. The protein expression of DLC1 was decreased about 20% in H1299 cells that overexpressed miR-200c (Fig. 3B and C).

The results of the present study show that miRNA-200a regulates *ATRX*, *DLC1* and *HFE*; miRNA-200b regulates *DLC1*, *HFE* and *HNRNPA3*; miRNA-200c regulates *ATRX*, *DLC1* and *HFE* (Table II). The target prediction results from each of the four bioinformatics algorithms are provided on our website (http://www.wvucancer.org/guoLab/Publications). There is a concordant downregulation of these potential targets at both mRNA and protein expression levels in the corresponding H1299 cells overexpressing the miR-200 family. All of the potential targets contain binding sites for the corresponding miR-200 family members according to the microRNA.org database (26), except for *ATRX*/miR-200a and *HFE*/miR-200a. The 3′ UTR region of *DLC1* contains 2 binding sites for miR-200a, 3 for miR-200b and 3 for miR-200c. *ATRX* contains 1 binding site for miR-200c. *HFE* contains 1 binding site for miR-200b and 1 for miR-200c. *HNRNPA3* contains 3 binding sites for miR-200b (Fig. 3A). *HFE* is a predicted target of miR-200c, not miR-200a or -200b, in NSCLC. Although miR-200b and -200c have the same seed sequence, the correlation between *HFE* and miR-200b was not statistically significant (p=0.057) in SCC patient samples. Therefore, it was not initially selected as a predicted target of miR-200b in NSCLC. Nevertheless, *HFE* was shown to be regulated by miR-200a, -200b and 200c in metastatic H1299 cells. *AHNAK* and *E2F4* are predicted targets of miR-200a. However, miR-200a did not suppress the expression of these two genes in H1299 cells (Fig. 2A). Similarly, *UBA6* and *UBE2I* were not regulated by miR-200b in metastatic lung cancer cells as they were predicted to be. These results were summarized in Table II.

To further substantiate the regulatory effects of miR-200 on these lung cancer prognostic markers, SCC patient samples (n=57) (23,24) were screened to investigate the correlation between the miR-200 family and the mRNA expression of its predicted target genes as well as *ZEB1* and *ZEB2*. The results showed that all the downregulated genes had a significant negative correlation with the corresponding miR-200 family member in SCC patient tumor tissues, except *ATRX*/miR-200a and *HFE*/miR-200a (Table III). The results in the patient samples further strengthened the *in vitro* findings.

**Figure 4:**
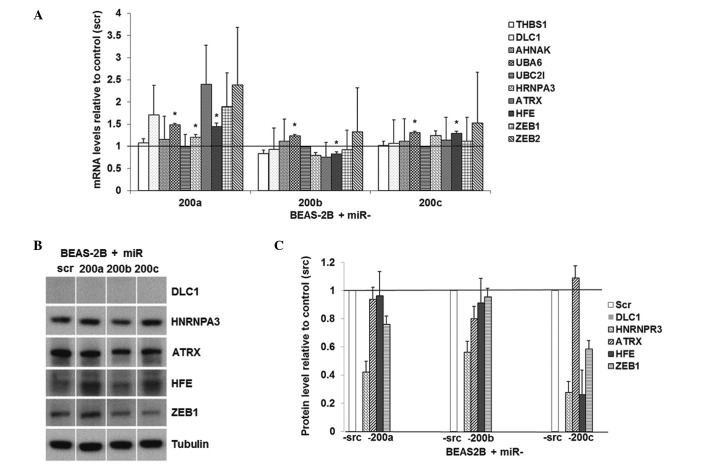
Relative mRNA and protein levels of the predicted molecular targets of miR-200 in BEAS-2B. (A) Relative mRNA expression of predicted miR-200 target genes in BEAS-2B cells infected with miR-scrambled and miR-200a, -200b and -200c. (B) Western blot analysis of protein levels in BEAS-2B cells overexpressing miR-200a, -200b, -200c or miR-scr. One representative blot is shown. The experiments were repeated in three biological replicates. Tubulin was used as a loading control. (C) Semi-quantitative analysis of protein levels relative to negative control miR-scr. Protein level was determined as described in Materials and methods. DLC1 protein was not detected in BEAS-2B in western blots.

The regulation of miR-200 on these predicted target genes was also evaluated in human immortalized lung epithelial cells BEAS-2B. The overexpression of miR-200b in these cells resulted in significantly downregulated mRNA level of *HFE* (Fig. 4A). The overexpression of miR-200a, -200b and -200c in BEAS-2B cells caused approximately 60, 40 and 70% downregulation of *HNRNPR3* protein, respectively (Fig. 4B and C). The overexpression of miR-200b in BEAS-2B resulted in a 20% downregulation of *ATRX* at the protein level and the re-expression of miR-200c resulted in a 70% downregulation of *HFE* at the protein level. These results indicate that miR-200 family downregulates *HNRNPR3*, *HFE* and *ATRX* in normal lung epithelial cells. Together, these results substantiate the role of miR-200 family and its regulated genes in lung cancer initiation and progression.

### E-cadherin and miR-200

After the potential molecular targets of the miR-200 family were shown in the present study, we sought to explore the effect of miR-200s on EMT in the metastatic NSCLC cells. Re-expression of miR-200c induced a 1.53-fold upregulation of E-cadherin (*CDH1*) through the downregulation of the E-cadherin repressor transcriptional factors *ZEB1* and *ZEB2*, which is consistent with previous studies in breast cancer or NSCLC cells (16,30,31) (Fig. 2B). E-cadherin protein was highly expressed in normal lung SAEC cells, but not in metastatic NSCLC H1299 cells (data not shown; http://www.wvucancer.org/guoLab/Publications). These results indicate that re-expression of miR-200 may reverse EMT process.

### Molecular network analysis

**Figure 5:**
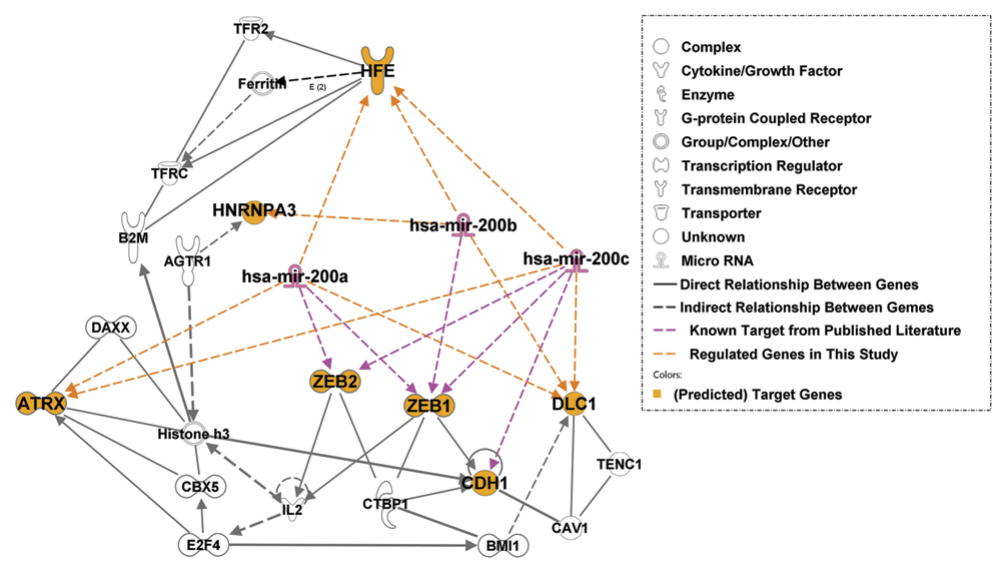
Molecular network analysis of the miR-200 family and potential molecular targets with Ingenuity pathway analysis (IPA).

Molecular network interactions and significant canonical signaling pathways associated with miR-200s and their predicted molecular targets were retrieved using IPA. The molecular network map shows interactions between the miR-200s and their known target genes, *ZEB1* and *ZEB2*, as well as potential targets identified from the present study (Fig. 5; the regulation identified in the present study is shaded in orange font). Among the genes in the molecular network, *Histone H3* and E-cadherin (*CDH1*) were major focal points in the miR-200 network. *Histone H3* is a component of the nucleosome and *CDH1* is a cell adhesion protein and epithelial phenotype marker. These results suggest that miR-200s and their potential target genes participate in molecular interactions involved in gene transcription regulation, either during the regulation of gene expression at the chromatin level or in the regulation of cell-cell interactions as mediated by E-cadherin. Increasing evidence indicates that chromatin remodeling induced by DNA damage or epigenetic changes are responsible for carcinogenesis.

The IPA functional analysis found a total of 69 canonical pathways associated with the miR-200 network, of which 13 canonical pathways were statistically significant (adjusted p<0.05 with Benjamini-Hochberg tests; Table IV). The top signaling pathways include virus entry via endocytic pathways, allograft rejection signaling, OX40 signaling pathway, caveolar-mediated endocytosis signaling, communication between innate and adaptive immune cells, chronic myeloid leukemia signaling, molecular mechanisms of cancer and DNA double-strand break repair by homologous recombination, among others (Table IV). Furthermore, the IPA functional analysis found 25 significant diseases and disorders related to the miR-200 network. The top 3 diseases included genetic disorders, metabolic diseases and cancer (Table V). At the molecular level, beta-2-microglobulin (*B2M*), *CDH1*, *ZEB1*, *ZEB2*, *ATRX*, *HFE* and the *miR-200s* are involved in genetic disorders; *B2M*, *transferrin receptor 2* (*TFR2*), *HFE* and angiotensin II receptor (*AGTR1*) are involved in metabolic diseases; *B2M*, *E2F*, *miR-200s*, *ATRX*, *DLC1*, *ZEB1*, *ZEB2*, *CDH1* and caveolin 1 (*CAV1*) are involved in cancer (Table V). These results indicate that miR-200 network involves complex signaling pathways and mechanisms, and has implications in numerous human diseases and disorders.

## Discussion

Lung cancer is a dynamic and diverse disease and is associated with numerous somatic mutations, deletions and amplification events. Tumor recurrence and metastasis causes lethality and failure in lung cancer treatment. About 35–50% of stage I NSCLC patients will develop and die from tumor recurrence within 5 years following surgery (32,33) and adjuvant chemotherapy of stage II and stage III disease has resulted in very modest survival benefits (34). Epithelial-mesenchymal transition (EMT) is a key process in tumor metastasis. Novel therapeutic approaches targeting EMT are needed to effectively prevent tumor recurrence and metastasis.

miRNAs are small non-coding RNAs that regulate gene expression via degradation or translational inhibition of target mRNAs. Importantly, one miRNA can regulate the expression of multiple genes because it can bind to its mRNA targets regardless whether there is perfect seed sequence complementarity (5). Our previous studies identified prognostic marker genes for NSCLC (19–21). The expression of these prognostic biomarkers was associated with metastatic potential in early stage NSCLC tumors. Identification of miRNAs that regulate multiple prognostic biomarker genes could shed light on the mechanisms underlying tumor metastasis and potentially provide the basis for the development of novel therapeutic targets to improve the clinical outcome.

Deregulated expression of miR-200 family members has been observed in multiple cancer types (15,16,29,30). Numerous studies showed that miR-200 family members regulate the EMT and cancer cell invasion by suppressing the expression of *ZEB1* and *ZEB2* genes (15,35). *ZEB1* and *ZEB2* are key transcription factors regulating EMT by binding to an E box upstream of their target genes, which include E-cadherin, and repressing their expression. Moreover, *ZEB1* and *ZEB2* can repress the transcription of miR-200 genes via negative feed-back loop mechanism (15,36). Absence of E-cadherin in cell junctions renders loss of cell-cell communication, thereby allowing cancerous cells to acquire an aggressive, invasive phenotype. Thus, downregulation of E-cadherin is associated with increased lymph node metastasis and poor-prognosis of NSCLC (37). Numerous studies have reported a stringent control of E-cadherin expression by the miR-200s via suppression of *ZEB1* and *ZEB2*(16,30,31). The miR-200 family expression is lost in mesenchymal subtypes of epithelial cancers and negatively correlates with cancer cell invasion (15,16) and metastasis in NSCLC (38).

On the other hand, overexpression of miR-200 was also found in cholangiocarcinoma malignant cells compared to non-malignant cells (39), melanoma cell lines (29), ovarian cancer (40), colorectal cancer (41) and NSCLC (Fig. 1B). These results indicate that expression of miR-200 family during tumorigenesis is rather complex, and may adopt a bimodal pattern with elevated levels in primary tumors and dramatic downregulation in metastatic cells. Alternatively, the role of miR-200 may differ depending on cancer type and stage.

Despite strong evidence that miR-200s inhibit EMT and suppress cancer cell invasion, several functional overexpression studies have yielded conflicting results on the role of miR-200s in metastasis, supporting both their anti-metastatic (18,30,42–44) and pro-metastatic (45,46) potential.

The present study sought to determine whether some of our previously identified human lung cancer prognostic markers are potential molecular targets of miR-200a, -200b and -200c microRNAs. The study goal was to explore whether biomarkers associated with NSCLC poor prognosis are functionally involved in EMT and metastasis through miR-200 regulation. In order to identify new molecular targets of the miR-200 family, we used the H1299 NSCLC cell line. This cell line is *p53*-deficient, has metastatic characteristics and is devoid of miR-200 family and E-cadherin expression. We chose this cell model in order to identify new molecular targets following re-expression of miR-200a, -200b and -200c, independent of *p53* regulation (47–49). *p53* is a tumor suppressor protein that regulates the expression of a myriad of genes and miRNAs including the miR-200 family (47–50). The present study shows an increased expression of E-cadherin in H1299 cells re-expressing miR-200c, consistent with other published results (16,30). It has been shown that E-cadherin promoter is methylated in NSCLC cells (51) and re-expression of E-cadherin may not be solely dependent on suppression of *ZEB1* but also dependent on other processes such as promoter demethylation (52). The modest re-expression level of E-cadherin in the present study is in agreement with other reports (16,18,52), suggesting that more significant downregulation of *ZEB1* and *ZEB2* might be required for full re-activation of E-cadherin.

**Figure 6:**
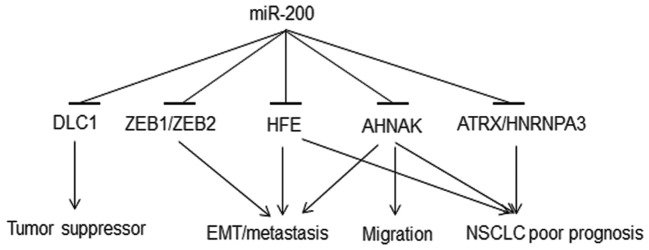
Proposed mechanisms of the miR-200 regulation in tumor initiation and metastasis.

This study identified a regulation of miR-200 family on their potential novel molecular targets. The results show that *DLC1*, *ATRX* and *HFE* genes are regulated by miR-200a and miR-200c; *DLC1*, *HFE* and *HNRNPA3* are regulated by miR-200b. Although, the changes in the expression of these genes at the mRNA and protein levels after re-expression of miR-200s in H1299 cells were relatively small (<2-fold), such small changes in the expression of microRNA targets are very common (16,53). Despite restoration of normal miR-200 levels, some other components of the post-transcriptional gene silencing pathway in H1299 cells might be in limiting amounts, for example Dicer, which is commonly downregulated in cancer cells (54). These findings are consistent with the model that miR-200s regulate their targets differentially either by targeting mRNA for degradation or/and by inhibiting its translation. All of these potential novel molecular targets of the miR-200 family are prognostic biomarkers for NSCLC (19–21). These genes showed overexpression in metastasis-prone NSCLC cells (H1299) compared with normal lung epithelial cells (NHBE and SAEC; Fig. 4B). *HFE* is important in iron metabolism disorder and oxidative stress (55). *HFE* polymorphism is associated with multiple cancer types and chemoresponse (56). The complex of beta-2-microglobulin (*B2M*) and its receptor HFE activates EMT and promotes metastases in human prostate, breast, lung and renal cancer cells both *in vivo* and *in vitro*, through the modulation of iron responsive pathways (57). Inhibition of either *B2M* or *HFE* reverses EMT (57). Our results show that *HFE* is regulated by all three of the studied miR-200 family members, indicating new mechanisms in EMT induction and lung cancer metastasis. *AHNAK*, a pseudopod-specific protein, also controls EMT in metastatic cancer cells (58). *AHNAK* knockdown in metastatic cells causes reduced cell migration and induces mesenchymal-epithelial transition (MET). Consistent results were observed in clinical cohorts, in which overexpression of *AHNAK* was associated with poor prognosis of NSCLC (19). Overexpression of *ATRX*(19) and *HNRNPA3*(59) was observed in NSCLC tumors and poor prognosis patients. Collectively, loss of miR-200 could lead to overexpression of *HFE*, *AHNAK*, *ATRX* and *HNRNPA3*, which in turn is associated with poor prognosis of NSCLC (Fig. 6).

*DLC1*, a tumor suppressor gene, is frequently silenced in various types of human cancer (60). *DLC1* was first identified in primary human hepatocellular carcinoma, with an inhibitory effect on the growth of breast and liver tumors (61). Downregulation of *DLC1* by miR-200a in primary human liver cells has been previously reported (62). In the present study, re-expression of miR-200a, -200b or -200c in metastatic human NSCLC cells resulted in *DLC1* downregulation at both mRNA and protein expression levels, with miR-200a exerting the most significant repression of *DLC1*. The overexpression of miR-200 family observed in primary tumors (Fig. 1B) could downregulate *DLC1*, which is involved in tumorigenesis (Fig. 6). Downregulation of *DLC1*, in turn, is associated with poor prognosis of NSCLC (21). These results, again, indicate potential pleiotropic regulatory mechanisms of miR-200 in lung cancer development and progression. Together, these results indicate that deregulation of miR-200 induces aberrant expression of multiple genes involved in lung cancer carcinogenesis, EMT, cell migration and metastasis, with significant implications on NSCLC clinical outcome. The proposed mechanisms of miR-200 regulation in carcinogenesis and metastasis are illustrated in Fig. 6.

IPA functional pathway analyses found that the miR-200 molecular network involved canonical pathways of immune response, molecular mechanisms of cancer, metastasis signaling transduction, cell-cell communication, proliferation and DNA repair. These results indicate that miR-200 is essential in regulating signaling pathways responsible for many biological functions and complex molecular mechanisms (Table IV). Moreover, the miR-200 related molecular network is implicated in at least 25 human diseases and abnormalities, including genetic disorders, metabolic disease, cancer and reproductive system disease, among many others (Table V).

In conclusion, this study combined computational predictions and quantitative experimental validations to demonstrate that the miR-200 family regulates multiple NSCLC prognostic marker genes. The identified regulation, direct or indirect, provides important insights of possible microRNA regulatory mechanisms in EMT and lung cancer metastasis and lays a foundation for future functional analysis. These potential molecular targets, each with significant prognostic value in NSCLC patients, are involved in the regulation of gene transcription and signal transduction pathways. The findings of the miR-200 downregulation of *DLC1*, *ATRX*, *HNRPNA3*, *AHNAK* and *HFE* in metastatic human NSCLC cells and the proposed regulatory mechanisms in tumorigenesis and metastasis could provide the basis for the development of novel therapeutic approaches for the treatment of this deadly disease. In our future research, reporter luciferase assays with mutations at specific seed sequences will be carried out to validate the direct interaction of these genes downregulated by miR-200 family.

## Figures and Tables

**Table I.** The predicted target genes analyzed in this study.

Gene symbol	Gene name	Assay ID	Function	Pathway	Remarks
*AHNAK*	AHNAK nucleoprotein (desmoyokin)	Hs00225285_m1	Protein-protein binding	Signal transduction	NSCLC prognostic biomarker (19,20)
*ATRX*	Alpha thalassemia/mental retardation syndrome X-linked	Hs00230877_m1	Transcriptional regulator, chromatin remodeling	Transcription	NSCLC prognostic biomarker (19,20)
*DLC1*	Deleted liver cancer 1	Hs00183436_m1	Regulation of small GTP-binding proteins	Signal transduction	Tumor suppressor gene (60) and NSCLC prognostic marker (21)
*E2F4*	E2F transcription factor-4	Hs00608098_m1	Transcriptional factor, cell cycle, apoptosis	Transcription	NSCLC prognostic maker (19,20)
*HFE*	Hemochromatosis	Hs00373474_m1	Regulation of body iron metabolism	Iron metabolism	NSCLC prognostic biomarker (21)
*HNRNPA3*	Heterogeneous nuclear ribonucleo protein-A3	Hs00864845_s1	Cytoplasmic RNA binding and trafficking, protein binding	Signal transduction	NSCLC prognostic biomarker (19,20)
*THBS1*	Thrombospondin 1	Hs00962914_m1	Extracellular adhesive glycoprotein	Protein interaction	NSCLC prognostic biomarker (21)
*UBE2I*	Ubiquitin-conjugating enzyme E2I	Hs00163336_m1	Ubiquitin-activating protein	Protein degradation	NSCLC prognostic biomarker (19,20)
*UBA6*	Ubiquitin-like modifier activating enzyme-6	Hs00414964_m1	Ubiquitin-conjugation for protein degradation	Protein degradation	NSCLC prognostic biomarker (21)

**Table II:** Genes regulated by miR-200a, -200b and -200c in H1299.

Genes	*miR-200a*			*miR-200b*			*miR-200c*		
		
	Predicted	Downregulated		Predicted	Downregulated		Predicted	Downregulated	
		mRNA	Protein		mRNA	Protein		mRNA	Protein
*AHNAK*	•								
*ATRX*			✓				•	✓	
*DLC1*	•	✓	✓	•	✓	✓	•		✓
*E2F4*	•								
*HFE*			✓			✓	•	✓	
*HNRNPA3*				•	✓	✓			
*THBS1*							•		
*UBA6*				•					
*UBE2I*				•					
*ZEB1*	•	✓	✓	•	✓	✓	•	✓	✓
*ZEB2*	•		✓	•		✓	•		✓

•, The gene is a predicted target for the corresponding miRNA;

✓, downregulation at mRNA level;

✓, downregulation at protein level.

**Table III:** Correlation between the expression of miR-200 and its regulated genes in squamous cell lung cancer patient tumors (n=57).

Genes	*miR-200a*	*miR-200b*	*miR-200c*
*ATRX*	−0.0629	NA	−0.301^a^
*DLC1*	−0.313^a^	−0.374^a^	−0.496^a^
*HFE*	−0.193	−0.253^b^	−0.393^a^
*HNRNPA3*	NA	0.264^a^	NA
*ZEB1*	−0.426^a^	−0.458^a^	−0.484^a^
*ZEB2*	−0.395^a^	−0.379^a^	−0.382^a^

a Statistically significant at p<0.05. NA, gene not regulated by miR-200 in H1299.

b Borderline significant at p=0.057.

**Table IV:** Top 13 significant canonical pathways related to the miR-200 molecular network in Ingenuity pathway analysis.

Canonical pathways	P-value	Molecules
Virus entry via endocytic pathways	0.0002	B2M, CAV1, TFRC
Allograft rejection signaling	0.0019	B2M, IL2
OX40 signaling pathway	0.0025	B2M, IL2
Caveolar-mediated endocytosis signaling	0.0044	B2M, CAV1
Communication between innate and adaptive immune cells	0.0056	B2M, IL2
Chronic myeloid leukemia signaling	0.0071	CTBP1, E2F4
Molecular mechanisms of cancer	0.0102	DAXX, CDH1, E2F4
DNA double-strand break repair by homologous recombination	0.0191	ATRX
Lipid antigen presentation by CD1	0.0257	B2M
Antiproliferative role of TOB in T cell signaling	0.0355	IL2
Colorectal cancer metastasis signaling	0.0407	CDH1, E2F4
Role of CHK proteins in cell cycle checkpoint control	0.0447	E2F4
Cell cycle regulation by BTG family proteins	0.0468	E2F4

**Table V:** Top 25 significant disease and disorder functions related to the miR-200 molecular network in Ingenuity pathway analysis.

Disease and Disorders	P-value	Molecules
Genetic disorder	0.00004	B2M, CDH1, IL2, TFR2, ZEB2, ATRX, mir-200, CAV1, ZEB1, HFE, AGTR1
Metabolic disease	0.00004	B2M, TFR2, HFE, AGTR1
Cancer	0.00005	B2M, E2F4, mir-200, ATRX, ZEB1, DLC1, CTBP1, CDH1, BMI1, IL2, ZEB2, CAV1, TFRC, AGTR1
Reproductive system disease	0.00005	B2M, CDH1, BMI1, IL2, ATRX, mir-200, CAV1, TFRC, DLC1, AGTR1
Gastrointestinal disease	0.00021	B2M, CDH1, BMI1, IL2, mir-200, ZEB2, CAV1, TFRC, AGTR1
Hepatic system disease	0.00021	B2M, BMI1, IL2, mir-200, DLC1, AGTR1
Organismal injury and abnormalities	0.00021	IL2, AGTR1
Infection mechanism	0.00053	CTBP1, E2F4, IL2, CAV1, TFRC, ZEB1
Infectious disease	0.00053	CTBP1, B2M, IL2, CAV1, TFRC, AGTR1
Hematological disease	0.00122	B2M, CTBP1, E2F4, IL2, ATRX, CAV1, DLC1, AGTR1, HFE
Dermatological diseases and conditions	0.00138	CAV1, ZEB1, HFE
Inflammatory response	0.00138	B2M, CDH1, IL2, ZEB1, AGTR1
Ophthalmic disease	0.00138	ZEB1
Respiratory disease	0.00186	CTBP1, B2M, CDH1, IL2, mir-200, CAV1, AGTR1
Immunological disease	0.00247	B2M, DAXX, E2F4, CDH1, HNRNPA3, IL2, TFRC, DLC1, AGTR1
Antimicrobial response	0.00276	IL2
Cardiovascular disease	0.00276	B2M, IL2, CAV1, AGTR1
Inflammatory disease	0.00401	B2M, DAXX, CDH1, HNRNPA3, IL2, ZEB2, TFRC, ZEB1, DLC1, AGTR1
Connective tissue disorders	0.00501	B2M, DAXX, CDH1, HNRNPA3, IL2, TFRC, DLC1, AGTR1
Neurological disease	0.00550	BMI1, ZEB2, CAV1, AGTR1
Renal and urological disease	0.00550	B2M, CDH1, AGTR1
Skeletal and muscular disorders	0.00733	B2M, DAXX, CDH1, HNRNPA3, BMI1, IL2, TFRC, DLC1
Developmental disorder	0.00961	ATRX, ZEB2, AGTR1
Nutritional disease	0.01230	IL2
Endocrine system disorders	0.04720	AGTR1
